# Healing Process after High-Intensity Focused Ultrasound Treatment of Benign Skin Lesions: Dermoscopic Analysis and Treatment Guidelines

**DOI:** 10.3390/jcm13040931

**Published:** 2024-02-06

**Authors:** Jacek Calik, Tomasz Zawada, Torsten Bove, Piotr Dzięgiel, Anna Pogorzelska-Antkowiak, Jacek Mackiewicz, Bartosz Woźniak, Natalia Sauer

**Affiliations:** 1 Department of Clinical Oncology, Wroclaw Medical University, 50-556 Wrocław, Poland; 2 Old Town Clinic, 50-136 Wroclaw, Poland; barwoz91@gmail.com; 3 TOOsonix A/S, 2970 Hoersholm, Denmark; tomasz.zawada@toosonix.com (T.Z.); torsten.bove@toosonix.com (T.B.); 4 Division of Histology and Embryology, Department of Human Morphology and Embryology, Wroclaw Medical University, T. Chalubinskiego 6a, 50-368 Wroclaw, Poland; piotr.dziegiel@umw.edu.pl; 5 Department of Human Biology, Faculty of Physiotherapy, Wroclaw University of Health and Sport Sciences, 51-612 Wroclaw, Poland; 6 EsteDerm Private Dermatology Clinic, 43-100 Tychy, Poland; a.pogorzelska.antkowiak@gmail.com; 7 Department of Medical and Experimental Oncology, Institute of Oncology, Poznan University of Medical Sciences, 61-701 Poznan, Poland; jmackiewicz@ump.edu.pl; 8 Faculty of Pharmacy, Wroclaw Medical University, 50-556 Wroclaw, Poland

**Keywords:** dermoscopy, High-Intensity Focused Ultrasound, HIFU, skin lesion healing, seborrheic keratosis, vascular lesion, sebaceous hyperplasia, sebaceous nevus

## Abstract

**Background**: High-Intensity Focused Ultrasound (HIFU) has emerged as a precise and non-invasive modality for tissue ablation and healing. This study presents a detailed dermoscopic analysis of skin healing post-High-Intensity Focused Ultrasound (HIFU) treatment, focusing on common benign skin lesions, such as seborrheic keratosis, sebaceous hyperplasia, vascular lesions, and sebaceous nevi. **Methods**: Prior to HIFU treatment, a comprehensive assessment was conducted, integrating ultrasound scanning and clinical evaluations. The TOOsonix System ONE-M was employed for HIFU treatments, with parameters tailored to each lesion type. **Results**: A common pattern observed across all lesions includes initial whitening post treatment, followed by scab formation and the development of a pink area with reparative vessels. This study, however, highlights distinct differences in fibrosis patterns and healing timelines across different lesion types. Each lesion type exhibited unique fibrosis patterns post treatment. Flatter variants of seborrheic keratosis healed within a month, displaying hypopigmentation and reparative vessels, alongside a distinct lattice fibrosis pattern in more verrucous forms, which took about two months to heal. Sebaceous hyperplasia, characterized by rapid healing within three weeks, demonstrated fibrosis with pink areas and perpendicular white lines, concluding with a slight depression. Vascular lesions varied in healing time based on depth, with superficial ones showing whitening and crust formation, while deeper lesions had vessel occlusion and size reduction accompanied by concentric fibrotic bands. Sebaceous nevi presented the longest healing duration of three months, characterized by amorphous white-gray structures, scab formation, and the emergence of pink areas with branching vessels, leading to clear skin with reduced white lines. **Conclusions**: in conclusion, this meticulous clinical evaluation highlights the unique healing characteristics and timelines for each skin lesion type treated with HIFU. These insights are invaluable for optimizing follow-up assessments, identifying potential complications, and refining treatment protocols. By providing detailed insights into the healing timelines and patterns for different types of lesions, patients can be better informed about their post-treatment journey.

## 1. Introduction

High-Intensity Focused Ultrasound (HIFU) has emerged as a powerful and non-invasive treatment modality with the potential to revolutionize medical interventions, offering a precise and controlled means of tissue ablation and thermally induced healing [1]. The fundamental principle underlying HIFU is akin to using a magnifying glass to focus light, where a concentrated beam of ultrasound waves targets a specific point in space, termed the focal point [2]. This approach offers a highly selective form of treatment, causing minimal damage to surrounding tissues while achieving effective tissue ablation [3]. The remarkable aspect of HIFU lies in its ability to create highly controlled and localized thermal and mechanical effects [4,5,6,7]. Moreover, the utilization of HIFU in dermatology has expanded with the introduction of a 20 MHz HIFU system, enabling the delivery of therapeutic focal points at the dermal and epidermal levels with high precision [8]. This results in the selective destruction of targeted tissue volumes, leaving adjacent tissues largely unaffected [9]. Precisely regulating acoustic treatments at temperatures between 40 °C and 90 °C makes the system suitable for a variety of non-ablative and ablative applications in dermatological treatment [8]. The ability to tailor the treatment parameters, such as power, pulse duration, and focal penetration depth, allows for the customization of the treatment process, ensuring that the desired therapeutic outcome is achieved with precision [10]. 

HIFU has garnered increasing attention for its application in a wide range of medical indications, encompassing the treatment of both benign and malignant conditions [11,12,13]. This advancement opens the door to effectively address a range of dermatological and aesthetic conditions [14,15]. HIFU has already proven its effectiveness in many skin disorders, including cutaneous neurofibromas, seborrheic keratosis (SK), condyloma acuminata, common warts, and many others [7,9,16]. Although treatment of skin lesions with HIFU is becoming more common, there is still a lack of information regarding the healing process itself. It is known that HIFU treatment induces significant thickening of collagen and elastic fibers in the reticular dermis and less pronounced changes in the papillary dermis [17]. Some papers indicate that the immediate effects of HIFU treatment observed in the treatment field were local whitening and swelling in round areas [6]. At the end of the treatment, sometimes blister-like white swelling and sometimes thin scabs are observed [6,16]. Importantly, the healing process looks different depending on the type of lesions treated [18]. In this work, we, therefore, focus on a meticulous clinical and dermoscopic evaluation of the healing process of the most common skin lesions. While the effectiveness of HIFU in treating these skin lesions has been established, a significant gap in the understanding of the post-treatment recovery process remains. By delving into the nuances of healing for sebaceous warts, sebaceous hyperplasia, vascular lesions, and sebaceous nevi, we shed light on the specific changes and timelines involved in the healing process. This research not only contributes to a deeper understanding of the healing trajectories after HIFU but also has practical implications for healthcare professionals and patients alike. Knowledge of the expected post-treatment outcomes, including the timing and nature of changes, can help manage patient expectations and refine treatment protocols. It may also offer insights into optimizing the timing of follow-up assessments and identifying any potential complications or adverse effects. Our research aims to fill this gap by exploring the healing process for various skin lesions post-HIFU treatment. This study is driven by the necessity to optimize patient care and enhance treatment protocols based on specific lesion characteristics. Additionally, a significant aspect of our work is to improve patient education. By providing detailed insights into the healing timelines and patterns for different types of lesions, patients can be better informed about their post-treatment journey. This understanding is crucial in managing expectations, improving patient comfort, and ensuring satisfaction with the treatment outcome.

## 2. Materials and Methods

Subjects diagnosed with sebaceous keratosis, sebaceous hyperplasia, sebaceous nevi, and vascular lesions were prospectively recruited for our investigative analysis. In this study, the average age of the 28 female participants was 49.0 years, while the 10 male participants had an average age of 54.9 years. A comprehensive examination of 233 cutaneous anomalies was conducted, encompassing 164 instances of seborrheic keratoses, 37 hemangiomas, 23 cases of sebaceous gland hyperplasia, and 9 occurrences of sebaceous nevi within the scope of our study. 

Informed consent was obtained from all participants, and this study adhered to the ethical principles outlined in the Helsinki Declaration II. Patient follow-up included dermoscopic examinations one month post treatment.

Prior to HIFU treatment, an ultrasound scan using a Dermascan^®^C 20 MHz B-mode ultrasound imaging system (Cortex Technology ApS, Aalborg, Denmark) was conducted. The system’s integrated software allowed measurement of the depth of the lesions, aiding in the selection of the appropriate HIFU handpiece with the appropriate focal penetration depth. Additionally, a clinical evaluation of each skin lesion using the Fotofinder Medicam 1000 device (FotoFinder Systems GmbH, Bad Birnbach, Germany) was performed, during which the selected lesions for distinct characteristics relevant to their specific conditions were assessed.

HIFU treatments were conducted utilizing a System ONE-M (TOOsonix A/S, Hoersholm, Denmark) operating at a frequency of 20 MHz. The apparatus is equipped with an advanced, real-time optical monitoring system, integrating a digital dermoscopic camera in the handpiece to observe the treated region in non-polarized light. The amalgamation of high spatial resolution in the ultrasound field and concurrent real-time dermoscopy observed on the system’s user interface screen facilitated an accurate administration of therapeutic energy, ensuring precise dosing targeted at the designated locations on the lesions.

Before initiating HIFU treatment, the transducer chamber was filled with deionized (DI) water and sealed with polyethylene film. Acoustic coupling was achieved using Parker Aquasonic 100^®^ ultrasound gel (Parker Laboratories Inc., Fairfield, NJ, USA). The specific parameters for HIFU treatment varied based on the type of skin lesion. The TOOsonix ONE-M device offers four handpieces with focal penetration depths (location of the focal point below the skin surface) of 0.8, 1.3, 1.8, and 2.3 mm. The HIFU energy ranged from 0.7 to 1.3 J per single exposure, administered in a contiguous “shoulder-by-shoulder” fashion. Each dose had a duration of 150 ms. A spatial separation of 1 to 2 mm was maintained between consecutive doses to ensure comprehensive coverage of the entire lesion field while preserving a minimal circumferential margin of approximately 1 mm. The administration of each dose recurred at an interval of approximately 1–2 s. Real-time monitoring of the treatment’s progress and status was facilitated through the utilization of the integrated dermoscopic imaging system. It is noteworthy that, based on our previous experiences, we refrained from employing pre-treatment topical anesthesia or local intralesional injections.

## 3. Results

### 3.1. Seborrheic Keratosis

Sebaceous keratosis (SK), arising from classic seborrheic keratosis, presents as brown, well-demarcated plaques, within which parallel brown lines, often referred to as “fingerprints” or “fat fingers”, can be frequently observed. SK transforms into sebaceous warts as the number of degenerated keratinocytes increases to a point where they give rise to “brain-like structures”. These structures are characterized by depressions in the skin, surrounded by centrally located vessels, which may be displayed as curved or linear. The vascular arrangement may evoke the appearance of dots and is encircled by a white halo.

SK can be classified into various types: pedunculated sebaceous keratosis, sebaceous keratosis with a squat base, excessively keratinizing sebaceous keratosis, and pigmentary sebaceous keratosis (where melanin-filled keratinocytes lend a gray-blue hue), rendering them diagnostically challenging. SK can range from small lesions on a small base with a diameter of 0.1 mm or more and have a single shaggy convex structure to SK with a wider base, and these wart-like structures are much more numerous. Dermoscopically, SK typically presents a brown, structureless area with sharp demarcation, often so precise that they create patterns akin to “moth-eaten” concave areas. Within these homogenous brown regions, one may observe brown circles and curved lines. Horn pseudocysts filled with keratin are a common finding within SK, representing keratinous masses located in the epidermis.

The course of HIFU treatment is dependent on the specific type of SK. Optimal cosmetic results are achieved on smaller pedunculated SK. Treatment commences by delivering HIFU to the base containing blood vessels. Following the initial treatments, the keratosis contracts, becomes pallid, and loses its blood supply. Consequently, the keratosis transitions from a fleshy hue to whitish-gray. Subsequently, the lesion undergoes necrosis, forming a scab, which ultimately detaches. The area from which the scab has separated is characterized by hypopigmentation and the presence of numerous, primarily linear, reparative vessels.

**Figure 1:**
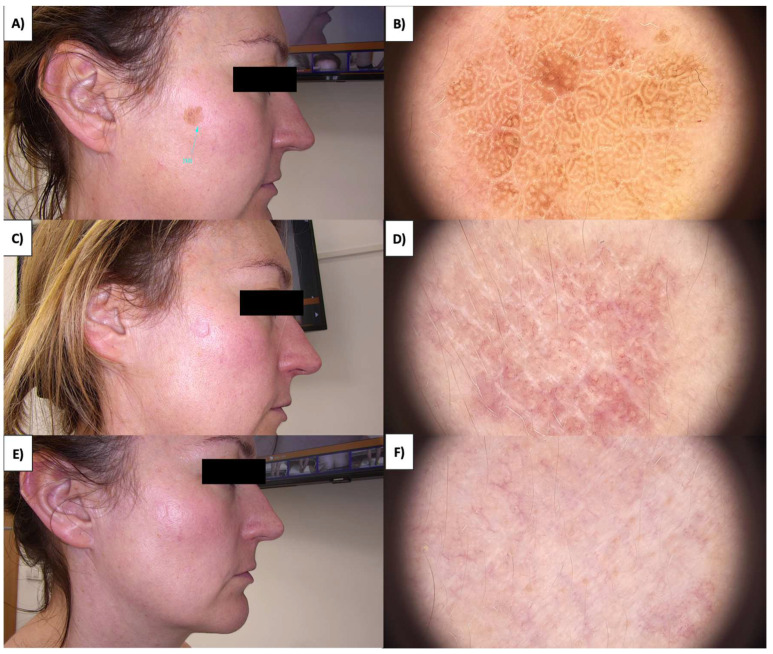
(**A**) SK before treatment—macroscopic view. (**B**) SK before treatment—dermoscopic view. (**C**) SK immediately after HIFU treatment—macroscopic view. (**D**) SK immediately after HIFU treatment—dermoscopic view. (**E**) Healing process of SK one month after HIFU treatment—macroscopic view. (**F**) Healing process of SK one month after HIFU treatment—dermoscopic view.

**Figure 2:**
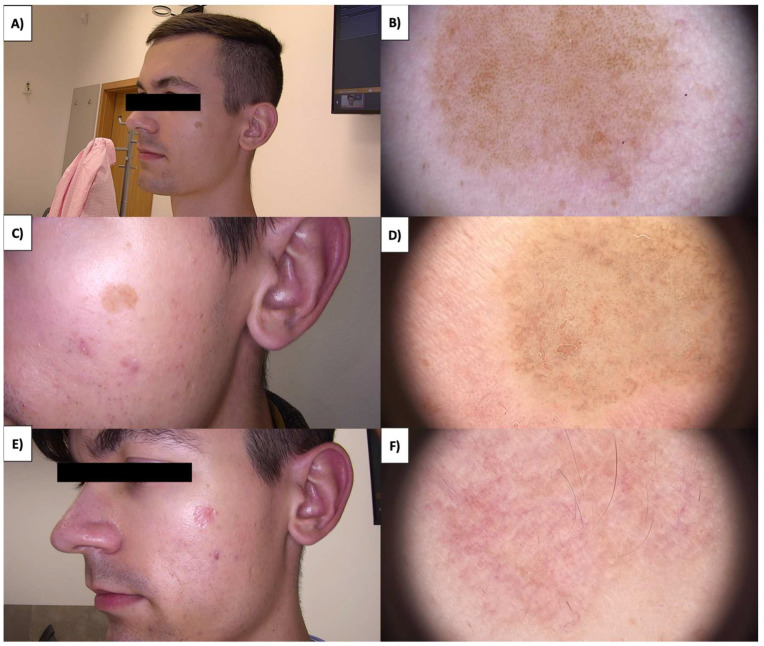
(**A**) SK before treatment—macroscopic view. (**B**) SK before treatment—dermoscopic view. (**C**) SK immediately after HIFU treatment—macroscopic view. (**D**) SK immediately after HIFU treatment—dermoscopic view. (**E**) Healing process of SK one month after HIFU treatment—macroscopic view. (**F**) Healing process of SK one month after HIFU treatment—dermoscopic view.

In the case of more sessile, less keratinized lesions, the treatment typically commences at the lesion’s periphery while preserving a small margin of healthy tissue (Figure 1 and Figure 2). Initially, the structures whiten, and a grayish granularity emerges, followed by scab formation. Upon detachment of the scab, pink, structureless areas with numerous tortuous vessels, often branching, looping, and occasionally displaying dot-like and reticular patterns, become evident. Interestingly, in these lesions during the healing process, an emerging pattern of lattice fibrosis can be observed (Figure 1D and Figure 2E).

In instances of highly keratinized lesions, ultrasound waves are frequently reflected by the keratinous mass during the procedure. Given that ultrasound cannot effectively penetrate heavily keratinized tissues, the removal process is typically conducted in multiple stages. Ultrasound is deposited in areas devoid of keratinization, leading to the onset of necrosis. As necrosis becomes apparent, a portion of the keratinization detaches, facilitating further treatment.

**Figure 3:**
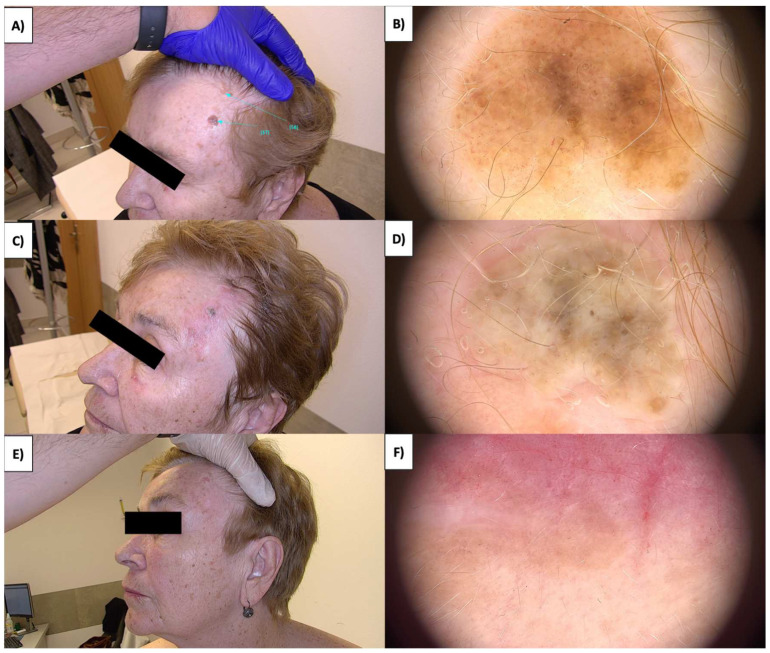
(**A**) SK before treatment—macroscopic view. (**B**) SK before treatment—dermoscopic view. (**C**) SK immediately after HIFU treatment—macroscopic view. (**D**) SK immediately after HIFU treatment—dermoscopic view. (**E**) Healing process of SK one month after HIFU treatment—macroscopic view. (**F**) Healing process of SK one month after HIFU treatment—dermoscopic view.

**Figure 4:**
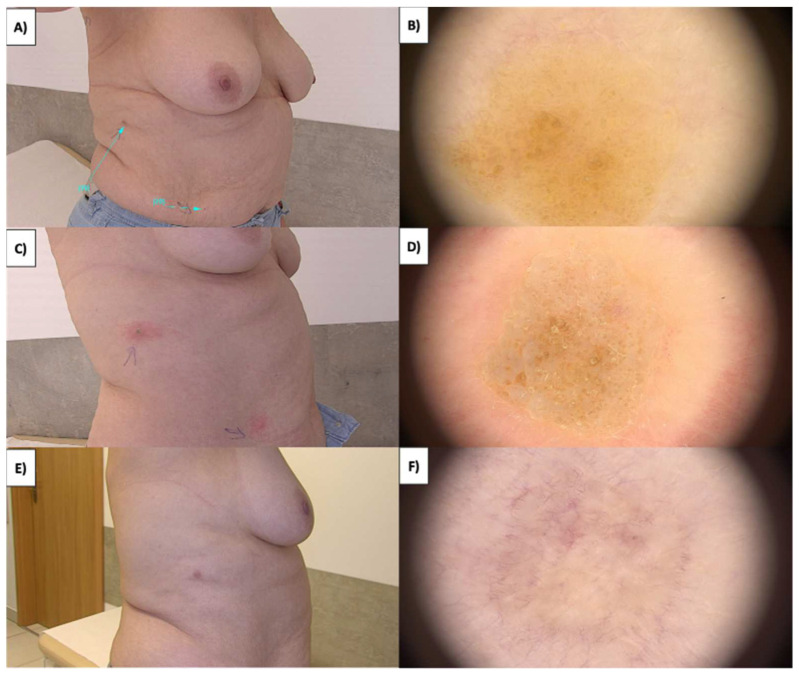
(**A**) SK before treatment—macroscopic view. (**B**) SK before treatment—dermoscopic view. (**C**) SK immediately after HIFU treatment—macroscopic view. (**D**) SK immediately after HIFU treatment—dermoscopic view. (**E**) Healing process of SK one month after HIFU treatment—macroscopic view. (**F**) Healing process of SK one month after HIFU treatment—dermoscopic view.

In cases of more protruding, nodular lesions, it is advisable to perform an ultrasound imaging assessment using an ultrasonic skin scanning device dedicated before the procedure. This allows for a precise evaluation of the lesion’s depth, enabling the selection of the appropriate handpiece penetration depth ranging between 0.8 and 2.3 mm. Typically, when dealing with deeper lesions, a stacked treatment may be beneficial. The procedure commences with the 1.8 mm handpiece followed by the 0.8 mm one. This sequence ensures that ultrasound waves initially traverse undamaged skin layers before penetrating deeper skin layers, inducing necrosis. In the second stage, the superficial layers are destroyed, facilitating a comprehensive removal of sebaceous warts in a single treatment. During the healing process, the pattern of fibrosis appears as an oval patch (Figure 3F and Figure 4F).

### 3.2. Sebaceous Hyperplasia

**Figure 5:**
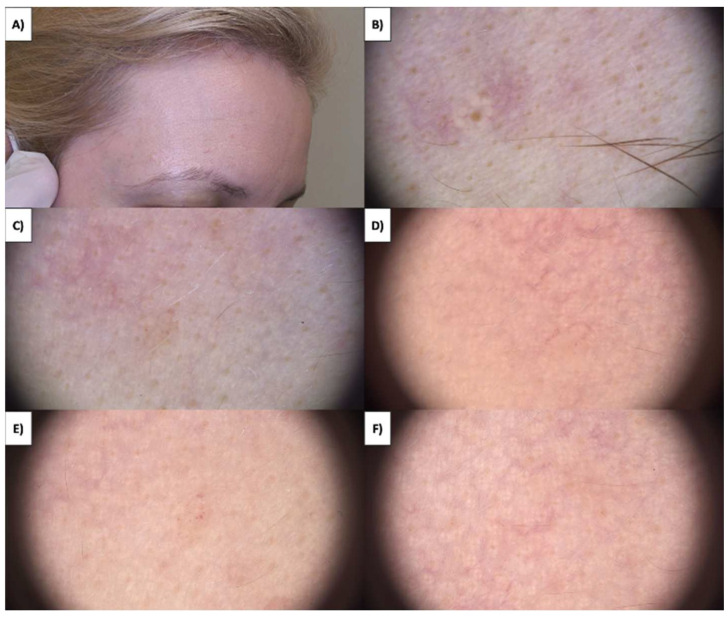
(**A**) SH before treatment—macroscopic view. (**B**) SH before treatment—dermoscopic view. (**C**) SH immediately after HIFU treatment—dermoscopic view with visible rosettes. (**D**) SH immediately after HIFU treatment—dermoscopic view with visible rosettes. (**E**) Healing process of SH one month after HIFU treatment—macroscopic view. (**F**) Healing process of SH one month after HIFU treatment—dermoscopic view.

Sebaceous hyperplasia (SH), the benign overgrowths of sebaceous glands predominantly located on the face and torso, appear as whitish-yellowish nodules encircled by blood vessels, forming a characteristic “crown” (Figure 5A,B). Notably, blood vessels never traverse the central portion of the lesion. As sebaceous glands are relatively superficial, HIFU treatment typically employs a 0.8 mm handpiece. Shortly after the procedure, an inflammatory response ensues, resulting in a pink area with reticular and linear branching vessels (Figure 5C,D). As a result of the procedure, white amorphous areas and white lines arranged perpendicular to fibrosis are often observed. Fibrosis in this context is minimal, owing to the small size of the lesion. Ultimately, during the course of sebaceous gland removal, a slight depression (tissue deficit) appears within one to three months, which gradually regenerates and returns to its original shape (Figure 5E,F).

### 3.3. Vascular Lesions

Vascular lesions, regardless of their location, most commonly exhibit a nodular arrangement of monomorphic vessels or a pattern of chaotic clusters of malformation-type vessels. These vessels can be found at various depths, ranging from the lower layers of the skin to the papillary layer. The HIFU procedure is performed following a prior assessment of the depth of these vessels. Therefore, HIFU treatment is most frequently conducted using a stacked treatment with two different handpieces with varying penetration focal depths.

**Figure 6:**
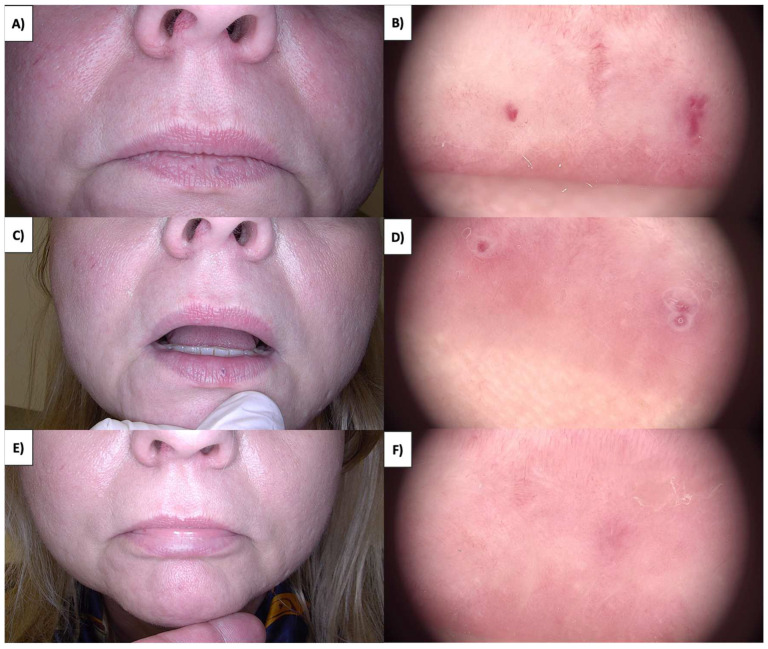
(**A**) Small capillary malformations in the lip—macroscopic view. (**B**) Small capillary malformations in the lip before treatment—dermoscopic view. (**C**) Small capillary malformations in the lip immediately after HIFU treatment—macroscopic view. (**D**) Small capillary malformations in the lip immediately after HIFU treatment—dermoscopic view. (**E**) Healing process of the lip one month after HIFU treatment—macroscopic view. (**F**) Healing process of the lip one month after HIFU treatment—dermoscopic view.

In the case of superficial lesions, such as those on the lips, where a nodular pattern is observed, the procedure results in whitening in the form of a homogenous, perfectly circular area with a grayish vessel at the central portion (Figure 6A–D). Shortly after, the lesion undergoes healing, the crust falls off, and punctate and linear vessels appear on a pink background. Over time, the number of vessels decreases, and the skin becomes completely clear (Figure 6E,F).

**Figure 7:**
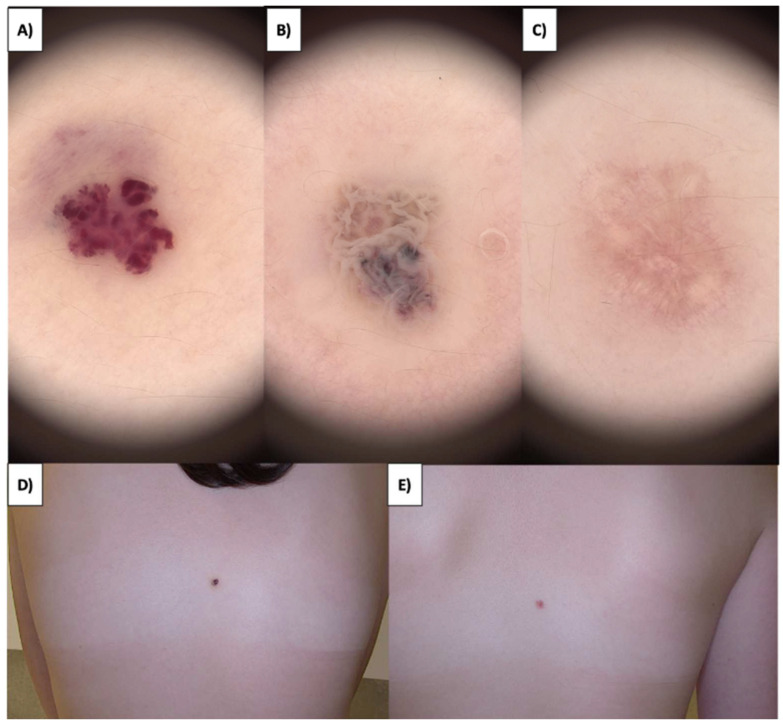
(**A**) Campbell angioma of the back before treatment—dermoscopic view. (**B**) Campbell angioma of the back immediately after HIFU treatment—dermoscopic view. (**C**) Healing process of Campbell angioma of the back one month after HIFU treatment—dermoscopic view. (**D**) Campbell angioma of the back before treatment—macroscopic view. (**E**) Healing process of Campbell angioma of the back one month after HIFU treatment—dermoscopic view.

In the case of deep lesions, there are instances where partial occlusion of the vessel occurs. The portion of the vessel that remains unoccluded results in a reduction in size. Nevertheless, the cosmetic outcome is highly favorable (Figure 7A–E). Importantly, during the healing process of the skin, the pattern of fibrosis resembles concentrically oriented bands converging toward the lesion (Figure 6F and Figure 7C).

In some cases, particularly when the skin is initially compromised, such as post radiotherapy, there is a phenomenon of depigmentation and closure of blood vessels, followed by relatively rapid revascularization of the vessels. The cosmetic outcome in such instances may be somewhat uncertain.

### 3.4. Nevus Sebaceous

**Figure 8:**
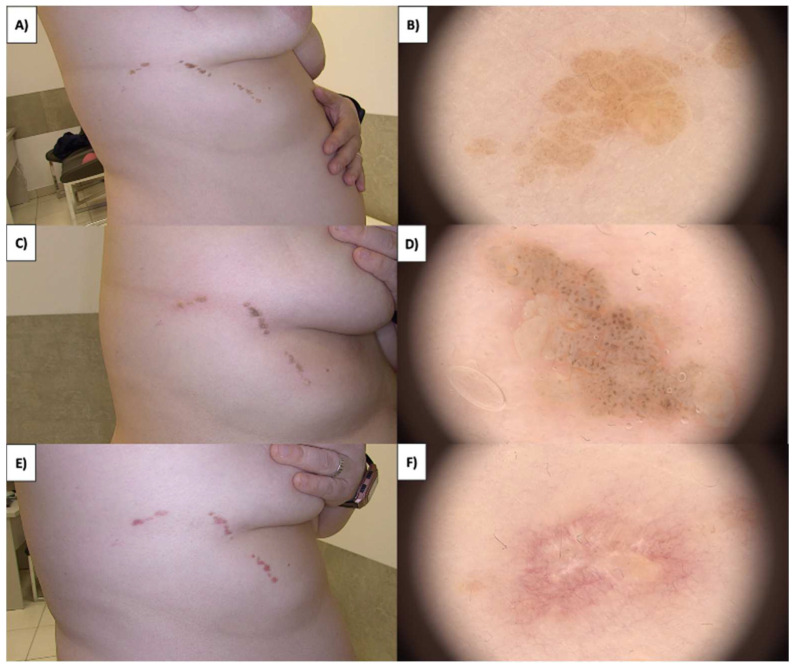
(**A**) NS—macroscopic view. (**B**) NS before treatment—dermoscopic view. (**C**) NS immediately after HIFU treatment—macroscopic view. (**D**) NS immediately after HIFU treatment—dermoscopic view. (**E**) Healing process of NS one month after HIFU treatment—macroscopic view. (**F**) Healing process of NS one month after HIFU treatment—dermoscopic view.

Sebaceous nevi (NS) are found on both the hairy scalp and the trunk’s skin (Figure 8A,B). They often present as extensive lesions and can manifest as early as childhood, sometimes even from birth. Over time, they develop a verrucous character with brown verrucous structures. An emergence of amorphous white-gray structures within the treated area is observed after HIFU treatment of NS (Figure 8C,D). Subsequently, a crust forms, and approximately one month post treatment, a pink area with branching linear vessels and short linear vessels appears (Figure 8E,F). Numerous multifaceted structures are observed in the fibrotic transformation process, as well as white lines and faintly demarcated areas with reflective properties under polarized light. The healing process takes about three months, during which there is a reduction in white lines and poorly demarcated areas, leading to clear skin with no therapeutic changes.

## 4. Discussion

HIFU is a method increasingly used in dermatology, which has recently gained tremendous interest [19,20,21,22,23]. To our knowledge, this is the first analysis focusing on the dermoscopic features observed during the healing period after HIFU treatment. Numerous studies provide evidence that HIFU at lower frequencies (1–5 MHz) affects skin rejuvenation [24,25,26,27]. Furthermore, HIFU offers advantages in the formation and reorganization of collagen, and a potential explanation for this effect could be an elevation in TGF-β levels along with the inhibition of MMP3 activity [24]. Furthermore, HIFU is recognized for its capability in non-surgical facial and body contouring, providing a safer and non-invasive alternative to traditional methods, effectively targeting fat tissue for contouring purposes in areas such as the abdomen, face, and neck [28]. Additionally, HIFU is known for its safety, with the most common adverse effect being transient pain during the procedure, while other significant adverse effects are notably rare, thus establishing it as a relatively safe option for skin treatment [29,30]. Importantly, the application of high-frequency HIFU, i.e., at 20 MHz, demonstrates an exceptionally high cure rate in treating premalignant lesions coupled with a favorable safety profile [23]. The HIFU treatment induces a targeted thermo-mechanical insult, typically accompanied by a wheal and flare response, reaching its peak after 5–10 min, and gradually subsiding over the following 10–30 min. Within a few days, a superficial dry wound may develop [23]. This wound tends to heal spontaneously over the course of several days or weeks, undergoing an initial phase of inflammation and potentially crustation. Notably, instances of HIFU-induced superficial dry ulceration or crustation resolve within a few weeks. Understanding the specific changes and timelines involved in the healing process contributes to a more holistic comprehension of HIFU’s dermatological applications. The outcomes of our exhaustive investigation furnish valuable insights into the healing kinetics subsequent to HIFU procedures for prevalent cutaneous lesions, including seborrheic keratosis, sebaceous hyperplasia, vascular lesions, and sebaceous nevi. 

**Figure 9:**
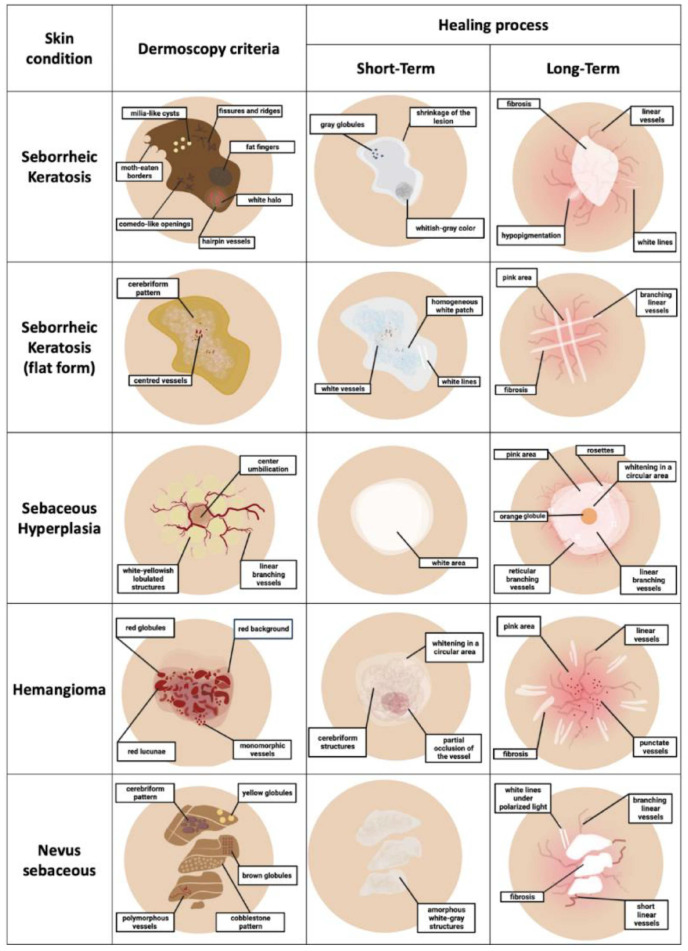
Summary description of the healing process after HIFU treatment.

Dermoscopically, seborrheic keratosis is identified by milia-like cysts, presenting as roundish white-yellow clods of variable size, corresponding to intraepidermal horn cysts [31,32]. Additionally, well-circumscribed oval or rounded black-to-brown clods, known as comedo-like openings, align with keratin aggregates in dilated follicular openings [33,34]. The distinct cerebriform appearance of SK is characterized by fissures and ridges—thick, curved lines with colors ranging from hypopigmented to brown, black, and blue. Exophytic papillary structures (gyri) are dome-shaped and closely juxtaposed, separated by blackish comedo-like openings [31]. Fat fingers, a variation of the cerebriform theme, manifest as thick, digitate linear, curvilinear, branched, or oval/circular dermoscopic structures [35]. Notably, demarcated borders and tiny light brown ridges running in parallel create a fingerprint-like pattern (Figure 9).

The vascular pattern of SK consists of hairpin vessels forming a half-looped or hairpin-like structure, often surrounded by a white halo—an established characteristic of keratinocytic neoplasms [34]. The distinct variations in the healing process of SK based on lesion type highlight the importance of tailored HIFU treatment approaches. Optimal cosmetic results are achieved when treating smaller pedunculated lesions, initiating the treatment at the lesion’s base containing blood vessels. The subsequent contraction, loss of blood supply, and transition to a whitish-gray appearance precede scab formation and eventual detachment [9]. 

**Figure 10:**
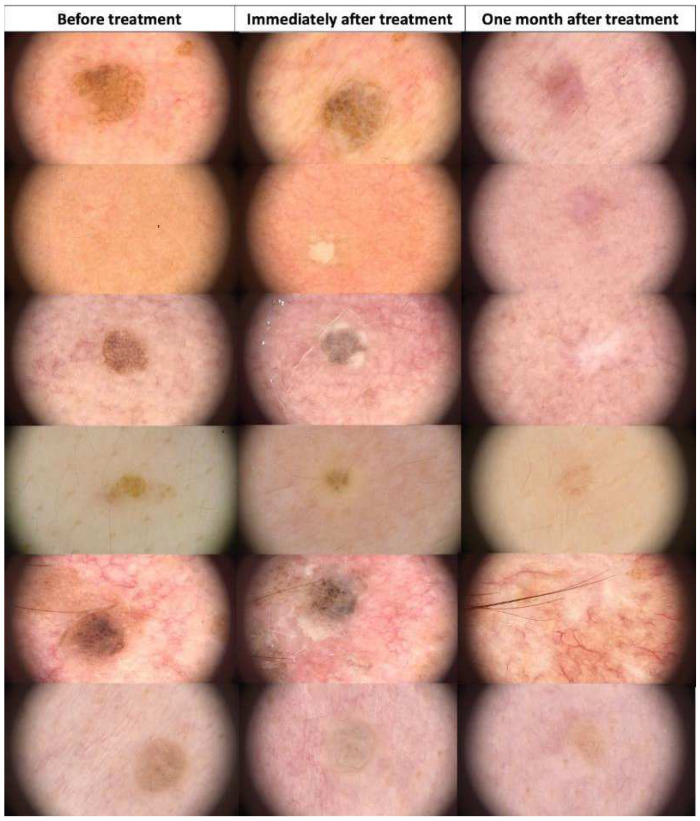
Dermoscopic view of seborrheic keratosis before HIFU treatment, immediately after, and after one month.

The healing process following HIFU treatment on seborrheic keratosis exhibits distinctive stages. Immediately after HIFU treatment, the SK displays an immediate circular whitening effect, extending approximately 2 mm around each targeted dose. This effect is attributed to the denaturation of proteins within the affected cells and a partial release of the epidermal layer, induced by the rapid increase in local temperature. For larger treatment areas, mild erythema, or redness, appears in the surrounding region due to histamine release, resembling an urticarial reaction. This reaction gradually diminishes over the subsequent 1–2 h. Even in instances where the very shallow focal depth of the selected handpiece results in a break in the basement membrane, no bleeding is observed, and the treated area remains dry and intact (Figure 10).

**Figure 11:**
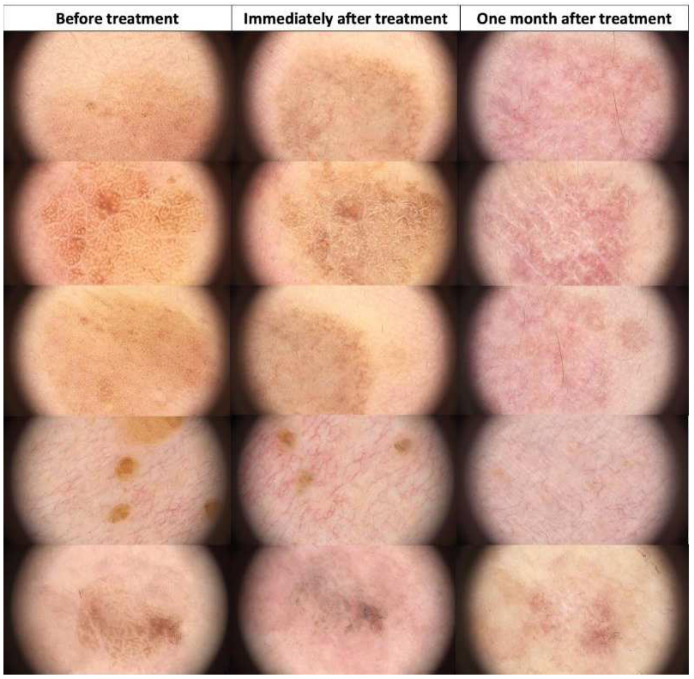
Dermoscopic view of seborrheic keratosis (flat form) before HIFU treatment, immediately after, and after one month.

The healing process involves distinct characteristics such as shrinkage of the lesion, gray globes, and whitish-gray color. For sessile, less keratinized lesions, the initial whitening and gray granularity also yield white vessels (denatured during the procedure). Over the next few days, a scab forms, which, when detached, reveals pink areas with branching linear vessels. In the case of strongly keratinized lesions, there is an additional manifestation of hypopigmentation, while the pattern of fibrosis forms into large oval patches (Figure 11). Conversely, for sessile, less keratinized lesions, the fibrosis pattern resembles a lattice-like structure. Importantly, we found that highly keratinized lesions present a distinct challenge necessitating a phased methodology, attributed to the reflective properties of ultrasound. The judicious deployment of probes featuring diverse diameters is instrumental in achieving optimal depth penetration and necrosis induction. In instances involving elevated nodular lesions, a preliminary ultrasound imaging assessment is instrumental in accurately gauging lesion depth and guiding probe selection. This customized approach serves to guarantee thorough lesion removal in a singular treatment session.

**Figure 12:**
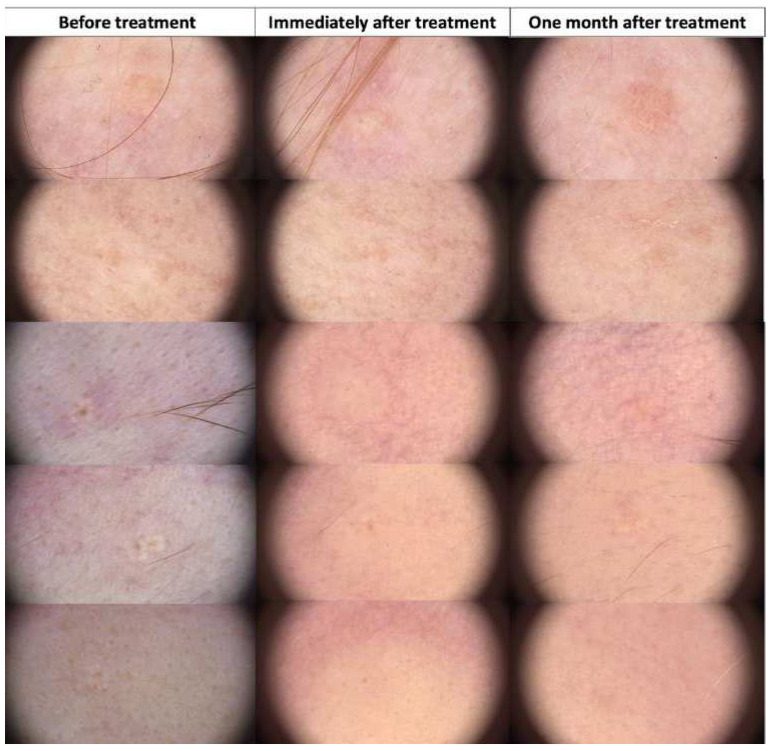
Dermoscopic view of sebaceous hyperplasia before HIFU treatment, immediately after, and after one month.

Dermoscopy features of sebaceous hyperplasia include aggregated white-yellowish globules or structures (cumulus sign) surrounded by crown vessels, characterized by groups of bending, scarcely branching blood vessels extending toward the center without crossing it [36,37]. Arborizing telangiectasias and the bonbon toffee sign, which involves a central umbilication or small crater surrounded by white-yellowish globules or structures, are additional criteria observed [37,38]. Milia-like cysts may occasionally be present (Figure 9). Sebaceous hyperplasia, characterized by nodules encircled by blood vessels, exhibits a distinctive “crown” pattern. Treatment with HIFU employing a 0.8 mm focal depth handpiece induces immediate whitening of the lesion (Figure 12). Over the ensuing days, a crust forms, revealing a pink amorphous area with whitening and an orange nodule at its center upon detachment. The fibrotic stage is not evident in this instance due to the small size of the sebaceous hyperplasia. Few regenerative vessels are observable, primarily linearly branched. Additionally, after treatment, rosettes become more pronounced. The procedure leads to eventual tissue deficit, which regenerates over time. This detailed understanding of the healing trajectory aids in managing patient expectations and refining treatment protocols.

**Figure 13:**
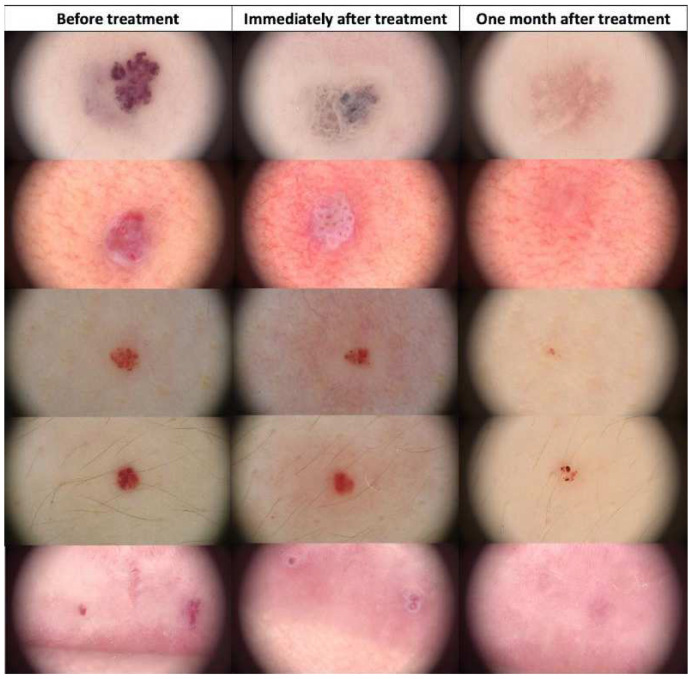
Dermoscopic view of hemangiomas before HIFU treatment, immediately after, and after one month.

Generally, hemangiomas manifest as red or dark superficial telangiectasia characterized by different degrees of capillary dilation, frequently accompanied by some level of atrophy and reduced skin elasticity [39,40]. Red lacunae are commonly observed and found in a significant portion of lesions. Background colors display diversity, with red being the predominant hue, followed by red-blue and red-white. Vascular structures beyond lacunae include linear curved vessels, serpiginous vessels, and coiled vessels (Figure 9). Lesions with multiple structures may display reticular, unspecific, or clustered vessel distribution [41,42]. It was previously shown that after HIFU treatment, a mild urticarial response occurred but resolved within hours [43]. Slight, transient redness persisted for months in the target area, gradually diminishing. In superficial congenital hemangioma, limited crust formation was observed, with internal systems facilitating cell transport. Protruding cherry angiomas quickly developed a dry crust, aiding external cell separation. In this work, we found that HIFU’s application in hemangiomas involves consideration of lesion depth. Superficial lesions exhibit homogenous whitening, followed by crust formation, vessel appearance, and eventual clearance. Deep vascular lesions may experience partial occlusion, reducing lesion size with favorable cosmetic outcomes. Over an extended healing period, the development of punctate and linear vascular structures becomes apparent, accompanied by fibrotic bands that orient centrally toward the treated area (Figure 13).

**Figure 14:**
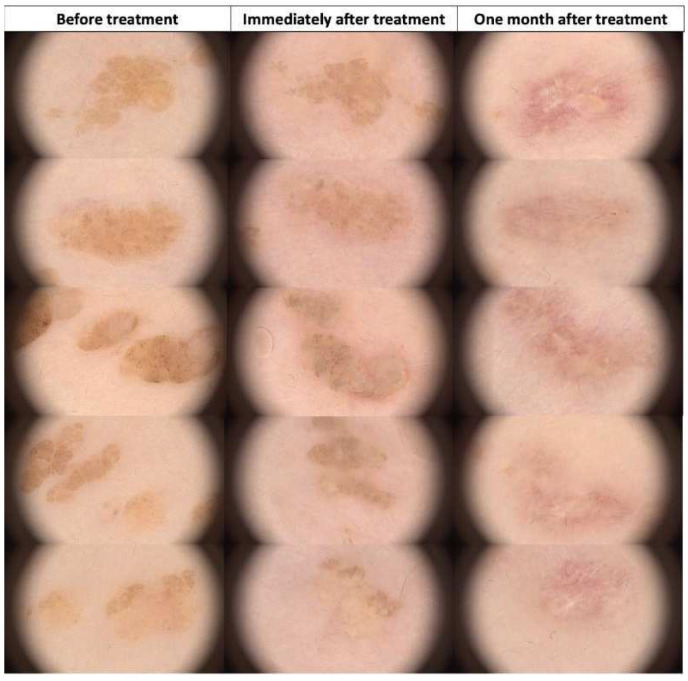
Dermoscopic view of sebaceous nevi before HIFU treatment, immediately after, and after one month.

The dermoscopic appearance of sebaceous nevi evolves with age [44,45]. In the infantile stage of sebaceous nevi, dermoscopy reveals clustered yellow globules on a yellow background or bright yellow dots without associated hair follicles. The childhood stage exhibits evolving lesions with cobblestone-patterned yellow globules corresponding to hyperplastic sebaceous glands. Unique orange-brown globules suggest a transitional phase, while brown globules indicate mature, hyperplastic sebaceous glands. In the adult stage with verrucous plaques, dermoscopy shows brown globules in a cerebriform pattern evolving toward the center alongside yellow nodules, indicating maximum epithelial hyperplasia (Figure 9). Secondary syringocystadenoma papilleferum associated with sebaceous nevi presents with predominantly grayish-white exophytic papillary projections, dotted vessels, erosions, crusts, and scales, reflecting the evolution of nevus sebaceus to syringocystadenoma papilleferum. Sebaceous nevi, frequently extensive and verrucous, undergo HIFU treatment, initially displaying amorphous white-gray structures that progress into a crust in the ensuing days. Upon crust detachment, linearly branched regenerative vessels and short linear vessels become apparent, along with the appearance of white lines under polarized light. Numerous multifaceted structures are observed in the fibrotic transformation process (Figure 14). The three-month healing period results in clear skin, emphasizing the importance of understanding the timeline and nature of changes for effective post-treatment management.

In our study, the treatments were carried out in a standard manner, focusing primarily on cosmetic aspects. It is important to note that we did not collect any numerical data on patient satisfaction. Our standard protocol for visits included monitoring the healing process and noting any complications, but it did not involve recording patient satisfaction scores. This limitation in our data collection method, specifically the lack of quantitative patient satisfaction measures, is acknowledged as a limitation of our study.

## 5. Conclusions

It has been found that healing times after HIFU treatment exhibit significant variability across different dermatologic entities. For seborrheic keratosis, the healing trajectory is notably diverse; the flatter variants typically resolve within a month, while the more verrucous types take about two months, indicative of the depth and complexity of their dermoscopic transformations and fibrotic patterns. Sebaceous hyperplasia, characterized by its superficial nature, demonstrates an expedited recovery process, usually concluding within three weeks. This rapid healing is largely due to the minimal fibrotic response observed in these lesions. Vascular lesions, regardless of their depth, tend to adhere to a uniform healing timeline of around one month, an important factor in monitoring their vascular characteristics and fibrotic evolution. The most prolonged healing phase is seen in sebaceous nevi, extending to approximately three months, attributed to their extensive and intricate dermoscopic changes. These variances in healing times are critical for clinicians in managing patient expectations and tailoring follow-up protocols.

This study contributes to the growing body of knowledge on High-Intensity Focused Ultrasound applications in dermatology, focusing on the dermoscopic evaluation of the healing process for common skin lesions. The dermoscopic features observed during the recovery period shed light on the intricate changes specific to each skin condition, offering valuable insights for healthcare professionals and patients. In cases of larger lesions treated with HIFU, there is a noticeable process of fibrosis that may lead to scar formation. Conversely, in smaller lesions, the skin typically heals cleanly, resulting in clear, healthy skin with no significant scarring. Understanding the nuanced healing trajectories post-HIFU treatment for seborrheic keratosis, sebaceous hyperplasia, hemangiomas, and sebaceous nevi is crucial for managing patient expectations and refining treatment protocols. The tailored approach, considering lesion characteristics and depths, ensures optimal cosmetic outcomes. Dermoscopic evaluations provide a detailed account of the short-term and long-term changes, offering practical implications for optimizing follow-up assessments and identifying potential complications. As HIFU continues to gain popularity in dermatological applications, this study contributes to the broader understanding of its efficacy and healing dynamics. The insights provided can guide healthcare professionals in optimizing treatment parameters and help patients make informed decisions about their dermatological care.

## Data Availability

No new data were created or analyzed in this study. Data sharing is not applicable to this article.
